# Use of a violence risk prediction tool (Oxford Mental Illness and Violence) in early intervention in psychosis services: mixed methods study of acceptability, feasibility and clinical role

**DOI:** 10.1192/bjp.2024.293

**Published:** 2026-02

**Authors:** Daniel Whiting, Margaret Glogowska, Sue Mallett, Daniel Maughan, Belinda Lennox, Seena Fazel

**Affiliations:** Institute of Mental Health, University of Nottingham, Nottingham, UK; Nottinghamshire Healthcare NHS Foundation Trust, Nottingham, UK; Department of Psychiatry, University of Oxford, Oxford, UK; Department of Primary Care Health Sciences, University of Oxford, Oxford, UK; Centre for Medical Imaging, University College London, London, UK; Oxford Health NHS Foundation Trust, Oxford, UK

**Keywords:** Precision medicine, psychotic disorders/schizophrenia, prognostic/prediction modelling, risk assessment, violence

## Abstract

**Background:**

Scalable assessment tools for precision psychiatry are of increasing clinical interest. One clinical risk assessment that might be improved by such approaches is assessment of violence perpetration risk. This is an important adverse outcome to reduce for some people presenting to services for first-episode psychosis. A prediction tool (Oxford Mental Illness and Violence (OxMIV)) has been externally validated in these services, but clinical acceptability and role need to be examined and developed.

**Aims:**

This study aimed to understand clinical use of the OxMIV tool to support violence risk management in early intervention in psychosis services in terms of acceptability to clinicians, patients and carers, practical feasibility, perceived utility, impact and role.

**Method:**

A mixed methods approach integrated quantitative data on utility and patterns of use of the OxMIV tool over 12 months in two services with qualitative data from interviews of 20 clinicians and 12 patients and carers.

**Results:**

The OxMIV tool was used 141 times, mostly in new assessments. Required information was available, with only family history items scored unknown to any notable degree. The OxMIV tool was deemed helpful by clinicians in most cases, especially if there were previous risk concerns. It was acceptable practically, and broadly for the service, for which its concordance with clinical judgement was important. Patients and carers thought it could improve openness. There was some limited impact on plans for clinical support.

**Conclusions:**

The OxMIV tool met an identified clinical need to support clinical assessment for violence risk. Linkage to intervention pathways is a research priority.

For some people with psychosis, identifying and reducing violence risk can form part of a prognosis-focused approach, given that violence perpetration can fragment care and support networks and is associated with poorer functional outcomes^1^ and stigma.^2^ It is also an adverse outcome with societal implications and economic costs.^3^ First presentation to clinical services is a higher risk phase of illness for violence.^4^ Risk is typically assessed in an unstructured manner, however, and clinicians can lack confidence and vary in their subjective weighting of clinical information.^5^ Resource-intensive assessment tools used by specialist forensic services are not feasible for non-forensic services, and so there is a lack of consistency and structured, practical support. Evidence-based scalable tools could improve assessment by augmenting and complementing clinical judgement. The Oxford Mental Illness and Violence (OxMIV) tool was developed with a focus on routinely available clinical information.^6^ It has performed well in external validation using pragmatic clinical predictor definitions and routine data from UK early intervention in psychosis (EIP) services, across a range of performance metrics (area under the curve (AUC) 0.75, sensitivity 71%, specificity 66%, with adequate calibration after updating). Notably, the OxMIV tool compared favourably on measures of net benefit with unstructured clinical judgement.^7^ To be useful, however, such new tools need to be implemented in practice. This study aimed to begin to bridge this translational gap to clinical use for the OxMIV tool by using mixed methods to examine (a) the acceptability of the approach to clinicians, patients and carers, (b) the practical feasibility and uptake of the tool and (c) its perceived utility, potential impact and optimal role within clinical pathways.

## Method

**Fig. 1:**
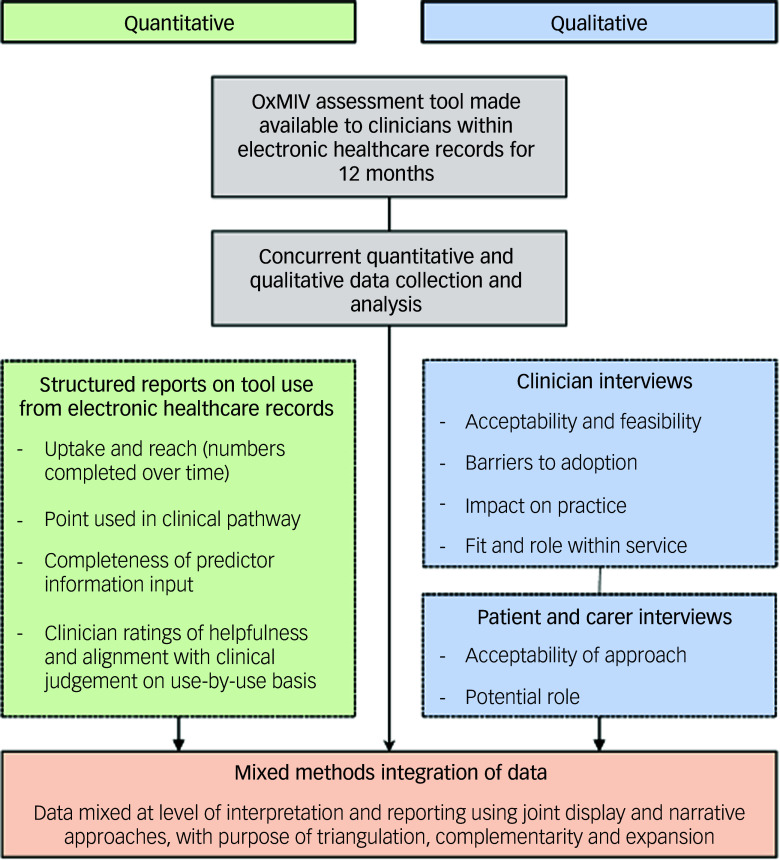
Outline of mixed methods study with concurrent qualitative and quantitative data collection. OxMIV, Oxford Mental Illness and Violence.

In two EIP services, we examined use of the newly implemented OxMIV risk assessment tool to support routine risk assessments over 12 months (July 2020–July 2021). Qualitative and quantitative data were collected concurrently and analysed separately in a convergent parallel design,^8^ with integration at the level of interpretation and reporting to reflect on combined meaning^9,10^ (Fig. 1). Joint display of quantitative and related qualitative results in a table to form an integrated results matrix was done to support the generation of meta-inferences (derived from integrating findings from both modalities).^11,12^

### OxMIV tool

The OxMIV model, openly published, combines 15 categorical predictors (e.g. previous drug misuse) and one continuous predictor (age)^6^ (Table 1) to calculate individual risk of violence perpetration within 12 months, presented as percentage risk and a categorical rating (low/increased). The OxMIV tool has been externally validated and updated (re-calibrated) using clinical data from UK EIP services to assess risk of violence perpetration resulting in police contact or involving a weapon or physical injury.^7^

**Table 1:** Predictors making up the Oxford Mental Illness and Violence tool and aligned definitions

Predictor	Definition	Data recorded
Age	Age at time of assessment.	Age in years
Gender	Sex at birth.	Male/female
Previous violent crime	Any lifetime conviction for violence (assault with or without injury, homicide, robbery, arson, any sexual offence, illegal threats or intimidation).	Yes/no
Previous drug misuse	Any lifetime diagnosis drug use disorder, from documented diagnosis or indicated by past contact with drug rehabilitation/treatment services or detailed history.	Yes/no
Previous alcohol misuse	Any lifetime diagnosis alcohol use disorder, from documented diagnosis or indicated by past contact with alcohol rehabilitation/treatment services or detailed history.	Yes/no
Previous self-harm	Any lifetime episode of self-harm (any form, e.g. self-poisoning, cutting).	Yes/no
Highest education	Formal schooling: secondary (to age 16), upper secondary (to age 18), post-secondary (past 18).	Secondary/upper secondary/post-secondary
Parental drug or alcohol misuse	Parental lifetime diagnosis of drug or alcohol use disorder (definitions as above for personal history).	Yes/no
Parental violent crime	Parental lifetime conviction for a violent offence (defined as above for personal history). History of incarceration taken as a proxy of violent offending.	Yes/no
Sibling violent crime	Sibling lifetime conviction for a violent offence (defined as above). History of incarceration taken as a proxy of violent offending.	Yes/no
Current episode	In-patient hospital admission or out-patient community patient at point of assessment.	In-patient/out-patient
Recent antipsychotic treatment	Prescribed and taken any antipsychotic drug in 6 months before assessment.	Yes/no
Recent antidepressant treatment	Prescribed and taken any antidepressant drug in 6 months before assessment.	Yes/no
Recent dependence treatment	Any pharmacological strategy to treat dependence (e.g. replacement therapy such as methadone) prescribed and taken in 6 months before assessment.	Yes/no
Personal income	Low: unemployed and/or inadequate financial situation with difficulty meeting basic daily living needs (food, accommodation etc.). Otherwise, ‘stable’.	Low/stable
Benefit recipient	Currently receiving social or disability benefits of any kind.	Yes/no

For the current study, the OxMIV tool was built into the risk assessment section of the electronic health record (EHR) for services in two English counties, Oxfordshire and Buckinghamshire (with a total catchment area of 1.2 million people). Pragmatic definitions for the predictors were specified (Table 1), complemented by a brief user guide. The OxMIV tool was assistive, not directive, that is, there were no directions with the risk score. Six of the predictors can be rated ‘unknown’ (leading to a risk range rather than a point estimate).

### Quantitative methods

We investigated the experience and utility of the tool. Data on use over 12 months following introduction were retrieved anonymously. This examined uptake (number completed), point used in the clinical pathway (interval between referral and OxMIV assessment completion) and availability of information (completeness of assessments and interval between when each OxMIV assessment was commenced/completed). For each assessment, clinicians categorically indicated (a) whether they had ‘identified any needs related to violence risk’ before completing the OxMIV assessment, and whether they deemed these needs ‘higher than for a typical patient’ in their service, (b) whether the OxMIV tool had ‘assisted in the overall assessment of needs in this case’ and (c) whether the OxMIV tool had ‘prompted any changes to careplans’. Fisher’s exact test^13^ was used to test for associations between categorical ratings. Whether the mean number of unknown predictors per assessment varied by pre-identified needs around violence was examined using Welch’s two-sample *t*-test.^14^ Quantitative analyses were undertaken with R version 4.1 for Windows (R Core Team, Vienna, Austria; www.R-project.org/),^15^ and visualisations with package ggplot2.^16^

### Qualitative methods

Data were collected using semi-structured individual interviews to examine the overall experience of the tool. Clinicians were recruited from the same EIP services using purposive sampling.^17,18^ Eligible clinicians were clinically qualified, with some role in violence risk assessment/management, who worked within the service during the relevant period. Target quotas were set according to professional background and duration of clinical experience. Recruitment continued until sufficient information power was achieved to address the study aims.^19^ Participants provided written informed consent.

Patients and carers were recruited from the same two services. Eligible patients and carers were male or female, aged 14–65 years, who were able to give written informed consent for participation (or written parental consent if aged 14–15 years), take part in an interview in English, were deemed suitable to participate by their usual clinician and had been involved in any reviews, assessments or discussions of risk subsequent to July 2020. Patient and carer participants received a £10 online shopping voucher.

For all participants, semi-structured interviews using topic guides were conducted by D.W., with length capped at 60 min. Transcripts were imported into NVivo 12 for Windows (QSR International; www.lumivero.com).^20^ Data were analysed thematically. Idea-by-idea open coding was built into wider categories that were refined as data were added and developed into themes discussed with the research team.^17^ Analysis was informed by the constant comparative method^21^ whereby collected data iteratively informed ongoing data collection.

### Public and patient engagement

A public and patient advisory group of five individuals with personal or carer experience informed study design, including topics for interview schedules, and reviewed participant-facing documentation

### Ethical considerations

The authors assert that all procedures contributing to this work comply with the ethical standards of the relevant national and institutional committees on human experimentation and with the Helsinki Declaration of 1975, as revised in 2013. All procedures involving human participants/patients in the OxMIV tool qualitative acceptability study of clinicians, patients and carers were approved prospectively by the West Midlands – Solihull Research Ethics Committee (reference 20/WM/0011). This was nested within a local project to improve violence risk assessment and documentation by making the OxMIV tool available within the EHR, approved by Oxford Health National Health Service (NHS) Foundation Trust’s quality and audit team. Quantitative data related to patterns of use of the OxMIV tool were obtained anonymously using the UK Clinical Record Interactive Search (UK-CRIS) system, approved by the local independent CRIS oversight group. Qualitative methods embedded core principles outlined by the Economic and Social Research Council.

## Results

In total, 141 OxMIV assessments were completed in 12 months (Supplement 1 available at https://doi.org/10.1192/bjp.2024.293). In *n* = 51 (36%), the clinician identified ‘any needs’ around violence risk before OxMIV assessment, and in 27 (19%) these were deemed ‘higher than average’. In parallel, 20 clinicians were interviewed from across professional backgrounds (Supplement 2). Six hours of interview dialogue was transcribed and analysed (average interview length 19 min). Clinician data were analysed across ten themes (Supplement 3). Twelve patients and carers were also interviewed (nine patients and three carers, Supplement 4), from which 4 h of dialogue was analysed (mean interview length 18 min). There were difficulties in recruiting patients with direct experience of the OxMIV tool, so interviews were amended to involve a more general discussion of the OxMIV tool.

### Integrated qualitative and quantitative findings

Table 2 presents integrated results where quantitative and qualitative findings examined the same clinical issue. To align with the study aims, these are considered together across the domains of (a) uptake, reach and role, (b) acceptability and availability of information and (c) utility and impact. Additional quotes are presented by theme in Supplement 3.

**Table 2:** Integrated results matrix combining qualitative and quantitative results examining the same clinical issue

Quantitative results	Qualitative results	Exemplar quotes	Analytical integration	Implications for practice and research
**Uptake, reach and role**				
141 OxMIV assessments undertaken in 12 months. OxMIV tool used with 40% of caseload or 65% of newly taken-on patients over 12 months. In 77% of OxMIV assessments the tool was completed the same day it was commenced.	***Clinician theme 3: practical usability and functionality within the electronic health record*** OxMIV took under 5–10 min and did not add significant administrative burden. Many felt questions were simple (no equivocation, drop-down boxes). Several clinicians felt main cognitive effort was coding predictors based on past diagnoses (e.g. drug use disorder). Option to score ‘unknown’ was helpful. Integration within health record system was deemed key to sustainable adoption. Many highlighted visibility, minimising duplication or navigation, and scope for auto-population.	‘It was trivially easy. The order that the questions are presented is logical and…it almost feels like a decision flow-chart that you’re going through. So…it’s presented in a very logical order whereby it asks you one question and naturally leads to the next.’ (P05, psychiatrist)	Convergent findings that OxMIV tool was efficient enough to have been used for a considerable proportion of risk assessments undertaken. Convergence across qualitative and quantitative findings that integration is a shift in approach that requires prompting and time to adopt. Subtotal understanding of purpose of tool amongst teams likely accounted for initial slow uptake. Prominence of tool on health record also relevant to uptake and level of awareness in team. Natural clinical role indicated by both sets of findings was as routine part of new assessment process (either first assessment or early phase of care coordinator contact) and used more selectively with patients already known to the team (i.e. only if concerns about risk).	Tool is feasible to use in EIP services. Requires time and repeated contact with clinical team during integration period. Basic purpose and process needs to be clearly communicated. Embedding of tool in health record important for future implementation. Future implementation should focus on routine role in new assessments.
There was a lag in uptake (Supplement 1), with 6% of total OxMIV assessments undertaken in the first 4 months. Rate increased when teams chose to focus on new assessments and incorporated OxMIV tool into routine administration.	***Clinician theme 7: barriers to integration and adoption*** Some initially unsure whether OxMIV tool globally applicable or only where active risk concerns. Ongoing processes for prompting use were felt necessary particularly with high staff turnover. Service is not primarily a risk management service, so discussion of formal risk assessment tools is not commonplace. The balance of attention given to risk management is important. Among clinicians who used the tool less, this was mainly because violence risk was perceived as low for patients they were supporting, and so not a priority.	‘I think…we’ve got these competing demands and I think we’ve really got to be careful that we do not hold risk management over and above…compassionate care. And…so we’ve got to…hold risk as a really important conversation. Something we need to respond to and be very aware of. But should not take precedence necessarily… I mean it’s a complex relationship…So obviously you want to be caring and risk is care for others as well, isn’t it?’ (P01, psychiatrist)		
OxMIV tool use was weighted towards early contact, with *n* = 92 (65%) of assessments undertaken within 12 weeks of referral.	***Clinician theme 6: positioning within the team and clinical pathway*** OxMIV tool fitted most naturally in the initial assessment phase, when risk is most fully considered. It aligned with the range of other baseline tasks/documentation.	‘…it felt right to do it after about 3 months when I’d gotten to know them a little bit more and get…more of a sense of their risk history. I would struggle to do it…very early on. If the risk assessment was fairly complete from before, then it…would be fine, but…the kind of thing that’s hard to ask about when you’re early in engagement. If they’ve ever had… convictions for example… I wouldn’t necessarily feel confident that I had all of the relevant information until I’d known them…for a couple of months.’ (P12, community psychiatric nurse)		
**Acceptability and availability of information**				
In 49 OxMIV assessments (33%) information was complete (Supplement 5). Mean 1.8 predictors (s.d. 1.6) scored as unknown per OxMIV assessment. Family history items scored unknown in 45–47% of assessments. Benefits, highest education and income category items scored unknown in 16%, 14% and 3%, respectively. Completeness of information did not significantly vary according to whether risk needs, or higher than average risk needs, had been identified before OxMIV assessment (*p* = 0.34 and *p* = 0.19 when compared to no risk needs identified before OxMIV assessment).	***Clinician theme 2: acceptability of the required clinical information*** Required information was considered clinically available and had face validity. A few clinicians were more reflective about the direct relevance of certain items for individual people. Many agreed that the family history items, and to a lesser extent educational history, were less routinely enquired about and required some thought as to their integration. ***Clinician theme 9: discussion and collaboration with patients*** Most clinicians continued to complete their risk assessment after, rather than during, clinical contact. Some felt that completing with a patient might add time or require a tactful approach. A few clinicians used OxMIV tool for discussion, such as about the impact of substance misuse on risk. The discussion of reducing risk was felt to be a more fruitful way that a collaborative approach could be pursued with OxMIV tool.	‘…I was intrigued about having…your highest education and also…parental violent crime and sibling violent crime… I hadn’t…thought in my head, I mean obviously the environment that somebody’s growing up in is…very…influential, but…it might be that yes their parents have done a violent crime but they…have no contact with them anymore or it might be…their siblings who they haven’t seen in 20 years… So although yes they…obviously…can be informative, they might not actually have a direct impact.’ (P03, occupational therapist)	Convergent findings that most information routinely available and acceptable. Family history less routinely explored in current practice. OxMIV tool did not seem to prompt gathering of extra information. Potentially explained by finding that some clinicians regard family history items as awkward to enquire about, and none typically completed assessment directly with patients, as well as some mixed views about relevance of all predictors in every case.	To improve information completeness tool may need to specifically suggest seeking additional information, highlight items’ relevance as predictors and reassure clinicians as to the acceptability to patients/carers.
**Utility and impact**				
Overall, *n* = 87 (62%) of OxMIV assessments were deemed to have been helpful by the clinician. This was more likely (*p* < 0.05) if they had pre-identified needs around violence risk (helpful in 75%) or above average needs around violence risk (helpful in 82%), compared to when no needs were pre-identified.	***Clinician theme 1: clinical utility and impact on practice*** The simplicity and clear focus was highlighted. Many described improved confidence, as OxMIV tool helped avoid omissions, aligned with clinical views, and supported clinical reasoning. Many clinicians stated OxMIV tool improved their knowledge of risk factors and embedded these in their wider practice. A few found confirming low risk helpful, including to summarise the multiple assessments in care records. ***Patients and carers*** Low risk rating from OxMIV assessment could be reassuring for families, who can feel sub-optimally informed by services because of confidentiality issues.	‘…it can be difficult to…remove any subjectivity from any risk so I think having [OxMIV] as well…helps perhaps do that. And so hopefully if the two marry up [OxMIV and clinical judgement], you can feel a bit more confident…about your risk assessment. And…then it kind of gives you…more confidence in using that as a rationale to…take it further. Because if for example you…wanted to take it to an MDT [team meeting] or a complex case or just to one of the consultants…you can…take it with…that as an added extra…to…support your…general risk assessment.’ (P04, community psychiatric nurse) ‘Definitely, definitely [would find communication of low risk from tool helpful]… it’s not ever a problem we’ve had, but it hasn’t stopped worrying about things that potentially could happen so you know when you’re lying there in bed at night and your imagination starts to run wild…’ (P2, carer)	Convergent findings that clinicians typically found OxMIV tool a helpful addition. Case-by-case utility expanded by qualitative descriptions of wider benefits. Some divergence, with qualitative findings highlighting merits of OxMIV tool when risk is low, whereas quantitative signal that more helpful when concerns about risk.	Findings support clinical use of OxMIV tool based on perceived utility, with low resource implications. Evaluation of impact needs to take account of the range of perceived benefits.
Among the 138 assessments where this information was available, there was 87% concordance between clinician ratings of above-average risk and the ‘increased risk’ category on OxMIV assessment (Cohen’s kappa 0.68, representing substantial agreement). When discordant, 78% (14/18) of assessments were rated as helpful, compared with 61% (73/120) of concordant assessments.	***Clinician theme 5: alignment and integration with clinical judgement*** All clinicians found OxMIV assessment typically aligned with their clinical view, and focused/clarified rather than altered it. Utility was not reliant on changing clinical view and it was important for acceptability that OxMIV assessment was not frequently discordant with clinical judgement. On some occasions clinicians felt risk was potentially higher than indicated by OxMIV assessment, typically because of lower-level aggression not captured by the tool.	‘…it’s got a good benefit to…time cost ratio…it’s not an hour long thing…it’s something you can do really quickly and a snapshot that…may not actually change your overall opinion…it might reassure your opinion you already had, or it might open up the discussion that will help your opinion…so on the back of it, even if it just leads to a discussion about “that’s a bit concerning, maybe we’ll discuss this in the team”, it kind of makes sense.’ (P08, social worker)	Convergent finding that changing the clinical view was not necessary for tool to be perceived as helpful. Simplicity of tool meant that it was perceived to offer net benefit.	Implementation of tool should highlight clinical role as a complement to clinical assessment that will more typically support/refine this clinical view, rather than contradict it.
Additional support was offered in 5–19% of cases (5% of all assessments, 12% of assessments where risk needs had been pre-identified and 19% of assessments where risk needs higher than average had been pre-identified). Changes were more likely where needs around risk had been pre-identified (*p* < 0.05).	***Clinician theme 10: attitudes towards linked interventions*** OxMIV tool did not typically lead to changes in careplans, largely as risks were low for most people. Many clinicians agreed that ideally risk assessment should be more embedded within subsequent management plans, and that this required more of a culture shift. The importance of such an approach remaining suggestive rather than directive was echoed by several clinicians. ***Patients and carers*** Several raised importance of assessment leading to support, and it is this aspect that patients and carers particularly want to be involved in.	‘…when we…risk assess for suicide risk, that always leads quite directly into…safety planning… Whereas risk of violence tends to be more…in our minds whether we need to think about MHA [Mental Health Act Assessment], or you know, something more restrictive…rather than…thinking more collaboratively. So…to have [suggestions for management] would be quite helpful.’ (P20, psychiatrist) ‘I think…all of this is about assessing the individual who has got a mental health issue, but…what support would be on offer to families where there is violence…so the follow-up support. If you discover the person has been violent…I would want something else to follow on from that, not just for the patient but for the family.’ (P9, carer)	Agreement that impact and benefit was in ways other than directing a practical addition to a careplan, although this occurred in 1/5 of those with the highest perceived risks. The idea of adding therapeutic suggestions to the tool was broadly palatable to clinicians, and important to patients/carers who wanted to be involved in this.	Tool would need to link to suggestions for management to have a more direct impact. A prompt to more directly involve patents/carers in the aligned treatment planning may be helpful.

OxMIV, Oxford Mental Illness and Violence; EIP, early intervention in psychosis.

### Other qualitative findings

Qualitative data also provided insights that were not directly examined by quantitative data.

#### Interpretation of output

Clinicians typically described using the categorical and probabilistic outputs of the tool in combination. The percentage score was a novel approach for most but was deemed easy to interpret. Several clinicians described this as more richly informative, by showing the magnitude of risk differences between individuals:

‘…low or moderate…is just one jump, whereas to look at a number…compared to somebody else you might’ve done the day before, it does show the big difference in the risk I think when…you’re not just jumping from one category to another. You’re looking at the difference in score completely…’ (P19, occupational therapist)

#### Stigma and labelling

Inadvertent stigmatisation or labelling was not found to be a concern. Many clinicians felt that whether risk was assessed formally with a tool or in the standard unstructured manner made no difference to such issues. The intended purpose of the tool for positive input and support was highlighted. One clinician was concerned that a tool focused on violence brought this to the forefront when not always relevant, and that integration within the general risk assessment processes could help this:

‘…if you’re doing a risk assessment then you’re already starting to…classify levels of risk and whether you…do it in the OxMIV way…with some numbers or you don’t…I don’t, personally…think it makes that much difference and… what we’re trying to do is…to be…as aware as possible of levels of risk so that we can help manage them…that’s potentially very much in the patient’s benefit if we can help them…manage their risks and not end up in trouble…then that’s gonna help them as well as others.’ (P15, psychiatrist)

#### Patient and carer perspectives

Patients and carers expressed positivity and thought the approach could improve openness. None would have objected to being involved in completing it during their assessment:

‘If you are feeling hesitant, a structure is always a useful way forward, isn’t it? And it also kind of opens up…other situations where there might be aggressive behaviour like…drugs or alcohol…So I think it’s good. It’s good to get it out in the open and it will cover more specific detail than somebody might disclose at the time…’ (P9, carer)

‘[OxMIV] looked pretty good…it’s easy to use, it was straightforward, the questions weren’t too invasive…they were put nicely.’ (P8, patient)

As highlighted in the integrated results, it was important to several participants that the assessment led to a helpful change in management. For patients, this was the aspect that they most wanted to be involved in, and carers highlighted that they also needed to be included. One carer described how worrying about potential violence was a stressful unknown around illness onset, and that the OxMIV tool could have a role in communicating the often-low magnitude of risk to families. This was in the context of all carers having some experience of feeling sub-optimally informed in their family member’s care, including risk, because of boundaries around confidentiality. One carer recounted a positive experience of interacting openly with services, where a frank discussion of violence risk was helpful in supporting the family during a crisis.

## Discussion

This mixed methods study examined the first use of a novel assessment approach (OxMIV tool) to support clinical violence risk management in 141 first-episode psychosis patients. Quantitative data on utility and patterns of use was complemented and expanded by qualitative interviews of 20 clinicians. Acceptability was further examined through qualitative interviews with 12 patients and carers. The integration of these methods yielded a detailed understanding of OxMIV tool adoption, its feasibility and acceptability and how the tool meets an identified clinical need to support risk assessments. As well as addressing the issue of why and how clinicians may choose to use the OxMIV tool to assist their decision-making, this work has implications for the clinical integration of other novel scalable digital tools, and how to study their implementation at scale in psychiatric settings.

### Acceptability and feasibility of the OxMIV tool

The OxMIV tool achieved good reach, being used in around 65% of new assessments. Clinicians found it straightforward, and it did not add significantly to administrative burden. Required information matched well with what was clinically available, with only family history items missing in many cases, reflecting clinical practice in not asking about this in standard assessments. Categorical predictors were well received. One reported cognitive effort for clinicians was considering diagnostic thresholds for previous drug or alcohol use disorders. This could be clarified in guidelines for the tool.

The OxMIV tool was also broadly acceptable from a service perspective. The balance between resource and benefits was thought to be favourable. There was also statistical concordance between the tool and clinical judgement, which was important for acceptability. Patients and carers stated that using a structured approach to improve assessments was acceptable, and could help with the openness of risk assessment.

### Uptake and barriers to use

Several findings were relevant to the resources and process required to integrate even a simple digital tool. There was a lag of 3–4 months between the tool’s availability and it being adopted to a notable degree. Clinicians highlighted how it was a novel addition for a non-forensic service. Another issue was varying awareness of whether it should be part of routine risk assessment for all, or only for individuals with significant forensic history. Promoting understanding required repeated communication, with staff turnover an additional challenge. Also, whilst the tool’s interface within the EHRs was acceptable, many thought that it needed fuller integration within general risk assessments. Among clinicians who did not use the OxMIV tool frequently, however, the main reason was that violence risk was a low clinical priority for their individual patient group.

One theme previously cited as a barrier to clinicians raising the topic of violence risk is stigma.^5^ However, in the current study, stigma was not felt to be a barrier to use of the OxMIV tool. There was consensus that this was no more a concern with the OxMIV tool than with other methods of assessing risk. Clinicians felt that uptake was correlated with the emphasis placed on violence risk within the team, which while important, is not considered their primary function.

### Utility and impact

Feedback from clinicians on a case-by-case basis was that the OxMIV tool assisted assessment in most cases, more so when there were existing concerns around violence risk. Some clinicians also found the reassurance of confirming low risk helpful. A clear finding was that helpfulness did not depend on the OxMIV tool altering the clinical view of risk. Clinicians spoke more around how it clarified and focused assessment, and that the percentage score provided richer information than categorical ratings by highlighting differences in the magnitude of risk. There were also broad benefits to confidence and knowledge. There was more limited impact in terms of the OxMIV tool prompting additional clinical support, although this was more common in those at highest risk, of whom around one in five had some extra clinical input (such as regarding substance misuse) incorporated following assessment.

### Clinical role and moving towards linked interventions

The OxMIV tool was preferred as part of the initial assessment, at first assessment and/or during the early period of contact with a care coordinator. By integrating it here, the OxMIV tool aligned with the information gathering and documentation already completed. This also aligns clinically in that people at first assessment are unknown to the service and are more likely to be actively unwell, making risk considerations timely. A secondary role was for refining risk assessments for those already known for whom there were concerns about violence risk. Clinicians saw less value in its use for those already known to them where risks were low, aside from providing a summary when documents had become unclear.

A strong theme from interviews with carers was feeling ‘in the dark’ around treatment because of confidentiality barriers, and carer participants were supportive of a tool as a way of framing their inclusion in conversations around risk. Rather than focusing solely on elevated risk, there was an identified role to reassure families where risks are low.

In line with their general approach to risk assessments, clinicians regarded the role of the OxMIV tool to assist formulation following assessment, rather than a tool to actively complete with patients. Some thought that in theory it could be used more directly, and patients and carers thought this would be acceptable. However, a more typical view was that collaboration could more helpfully be focused on developing a support plan in response to the assessment, rather than the risk assessment itself. This finding is in the wider context that collaboration in risk assessment is also limited in other settings, including forensic settings where it is a research priority.^22^

The limited impact of the OxMIV tool on what support clinicians offered is in keeping with previous studies of prediction models that suggest an assessment tool has less impact on its own than when presented with linked therapeutic recommendations.^23^ An example of this is a trial to reduce suicidal behaviour in people presenting to emergency departments with suicidal ideation or attempts that compared treatment as usual, universal risk screening and risk screening plus an intervention.^24^ Outcomes were only significantly better than treatment as usual in the intervention group. Similarly, in a cluster randomised controlled trial in forensic psychiatric out-patient settings, risk assessment alone did not reduce reoffending.^25^ Therefore, to directly influence interventions to reduce risk, the OxMIV tool will require linked treatment pathways. The acceptability of this was discussed, with clinicians responding positively, with the caveats that such guidance should remain suggestive rather than directive, to avoid care becoming protocolised. Further, patients and carers placed high value on the assessment leading to something helpful, and highlighted this as the aspect they most wanted to be directly involved in.

### Limitations

It proved difficult to recruit patients to interviews who had a significant violence history. Such individuals may have a different perspective on the issues explored. This difficulty seemed to mirror the wider perception by clinicians of violence being a sensitive topic, reported elsewhere, and they were perhaps more hesitant to invite those for whom it may be personally sensitive.

Work took place during the COVID-19 pandemic, which meant interviews were undertaken remotely, although conversely this may have facilitated discussion of more challenging topics. The service was also operating in a different fashion to usual. Finally, the researcher undertaking interviews became known to some of those in included services by working clinically during the project. To mitigate any risk of bias in interview responses, it was clarified at interviews that whether views of the tool were positive or negative had no implications for that researcher.

### Implications for future research

There are two main implications for future research. First, the study has shown the relevance of considering a prediction tool as a complex intervention in how it interacts with clinical systems,^26,27^ given the flexibility with which clinicians may integrate it, the range of behaviours that can be affected and the importance of systems and senior leadership for embedding sustainable use within a service. For example, in this study clinicians used the OxMIV tool in different ways, from a tool to summarise documentation and communicate with colleagues, to a way to discuss substance misuse with patients. They also described a range of impacts including on their wider practice, confidence and knowledge. Drawing on aspects of the framework for developing and evaluating complex interventions^28,29^ will therefore be important for future research on the clinical translation of prediction tools like the OxMIV tool, particularly in moving towards linking therapeutic interventions.

Second, this is a demonstration of how mixed methods can be used to examine clinical use of a prediction tool. In turn, this can also inform clinical translation work in psychiatry. This is a key area of need in the field, where a large gap remains between models developed and those translated into clinically impactful tools.^30^ A novel aspect developed here was harnessing the potential of EHRs^31^ to collect use-by-use structured feedback on utility and data on the patterns of use, and combining this with qualitative interviews. Integrating these sources of data provided clear added value and facilitated a more detailed understanding than any of these approaches would have provided in isolation. The contemporaneous use-by-use feedback also provides a perspective that is free from the recall bias that distal surveys may be prone to.

### Future for the OxMIV tool

For a prediction tool to be of potential clinical use, three broad areas need consideration: (a) addressing an identified clinical need; (b) the tool is sufficiently accurate in the target clinical setting; and (c) the tool is acceptable and feasible in that setting, with a defined clinical role. For the OxMIV tool, there is now evidence for predictive accuracy, acceptability and feasibility in clinical services for early psychosis. The current study substantially increases understanding of how the tool could be integrated clinically to address the challenges identified by previous work, and prevent violence outcomes,^5^ beyond simply increasing accuracy compared to unstructured judgement alone^7^ (Table 3). Future research should therefore focus on implementation studies as part of stepwise progress towards a more definitive trial or observational study of impact. For planning such work, the current study identified three specific considerations. First, a run-in period of several months is required for adoption, which needs inclusion in study designs. Second, a clearly specified role, such as for all new assessments, would need to be established by a sustainable process such as embedding within standard mandatory documentation. Once it is established within a team, use with 70% of all new assessments would be a realistic target. Finally, measuring impact of a risk assessment tool on a clinical service will likely need to consider the importance of outcomes that are more proximal than, for example, reductions in violent offending. This study has identified that examining the provision of specific additional support to reduce risk would be a candidate outcome. The importance of aspects such as team systems, administration and clinical leadership support were also well evidenced in the current study, and resources such as the Consolidated Framework for Implementation Research could provide a structure for future clinical implementation research.^32^

**Table 3:** Challenges of clinical violence risk assessment and possible solutions

Challenge of current practice based on prior work in early intervention in psychosis services^5,7^	Potential benefits from using OxMIV tool from this study
Clinicians have low confidence in violence risk assessment, lack training/knowledge of risk factors and perceive their unstructured judgements as subjective and informal. Weighting of risk information has been shown to vary in response to clinical vignettes.	Clinicians felt more confident that they were not omitting important information, and found the alignment of OxMIV tool with their overall clinical views reassuring. Clinicians stated OxMIV tool clarified/focused their assessments and meant they considered a broader range of risk factors.
Clinicians have varying views about relevance of static risk factors. Only half the patients with a previous violent conviction had violence risk noted as a relevant need in initial assessments.	Clinicians commented that having a percentage or probability score helpfully highlighted differences in magnitude of risk.
Clinicians have limited access to forensic history and are uncertain of the justification needed to seek this.	Clinicians felt their assessment was more robust and so they were more confident to discuss further, e.g. with seniors or other services.
Current interventions focus on crisis plans and practical safety measures.	Clinicians were open to development of an output from OxMIV tool that suggests approaches to address modifiable risk factors.
Risk assessment documents can become disorganised and often do not allow for an overall summary to be extracted.	Provision of a risk or probability score from OxMIV tool was a novel aspect and positively received. Even where risk was low, clinicians found it helpful to summarise prior risk assessment documents.
No standard way to document or communicate risk.	Clinicians found the categorical and percentage scores easy to interpret.
Clinicians are concerned about reinforcing stigma and the sensitivity of discussing violence risk.	None felt that OxMIV tool posed additional issues relating to stigma compared to standard risk assessment processes.

OxMIV, Oxford Mental Illness and Violence.

## Supporting information

Whiting et al. supplementary material Whiting et al. supplementary material

## Data Availability

Qualitative data that support the findings of this study (in the form of exemplar quotes) are available in the main text and supplementary material of this article. Additional qualitative data that support the findings of this study are available from the corresponding author, D.W., upon reasonable request. Quantitative data for this work is owned by Oxford Health National Health Service (NHS) Foundation Trust using anonymised patient records via CRIS powered by Akrivia Health. The data cannot be made publicly available but can be accessed with permissions from Oxford Health NHS Foundation Trust for UK NHS staff and UK academics within a secure firewall, in the same manner as the authors.

## Supplementary material

The supplementary material for this article can be found at https://doi.org/10.1192/bjp.2024.293

## References

[1] Cotton SM , Lambert M , Schimmelmann BG , Filia K , Rayner V , Hides L , et al. Predictors of functional status at service entry and discharge among young people with first episode psychosis. Soc Psychiatry Psychiatr Epidemiol 2017; 52: 575–85. 28233045 10.1007/s00127-017-1358-0

[2] Pescosolido BA , Halpern-Manners A , Luo L , Perry B. Trends in public stigma of mental illness in the US, 1996–2018. JAMA Net Open 2021; 4: e2140202. 10.1001/jamanetworkopen.2021.40202 PMC8693212 34932103

[3] Senior M , Fazel S , Tsiachristas A. The economic impact of violence perpetration in severe mental illness: a retrospective, prevalence-based analysis in England and Wales. Lancet Public Health. 2020; 5(2): e99–106. 32032564 10.1016/S2468-2667(19)30245-2 PMC7025880

[4] Youn S , Guadagno BL , Byrne LK , Watson AE , Murrihy S , Cotton SM. Systematic review and meta-analysis: rates of violence during first-episode psychosis (FEP). Schizophr Bull 2024; 50: 757–70. 38412435 10.1093/schbul/sbae010 PMC11283196

[5] Whiting D , Glogowska M , Fazel S , Lennox B. Approaches and challenges to assessing risk of violence in first episode psychosis: a qualitative interview study of clinicians, patients and carers. *Early Interv Psychiatry* 2024; 18: 1–9. 10.1111/eip.13502 PMC7618059 38356414

[6] Fazel S , Wolf A , Larsson H , Lichtenstein P , Mallett S , Fanshawe TR . Identification of low risk of violent crime in severe mental illness with a clinical prediction tool (Oxford Mental Illness and Violence tool [OxMIV]): a derivation and validation study. Lancet Psychiatry 2017; 4: 461–8. 28479143 10.1016/S2215-0366(17)30109-8 PMC5447135

[7] Whiting D , Mallett S , Lennox B , Fazel S. Assessing violence risk in first-episode psychosis: external validation, updating and net benefit of a prediction tool (OxMIV). BMJ Ment Health 2023; 26: e300634. 10.1136/bmjment-2022-300634 PMC10335427 37316256

[8] Creswell JW , Clark VLP. *Designing and Conducting Mixed Methods Research.* SAGE Publications, 2017.

[9] Fetters MD , Curry LA , Creswell JW. Achieving integration in mixed methods designs-principles and practices. Health Serv Res 2013; 48(Pt 2): 2134–56. 24279835 10.1111/1475-6773.12117 PMC4097839

[10] Fetters MD , Molina-Azorin JF. The journal of mixed methods research starts a new decade: the mixed methods research integration trilogy and its dimensions. J Mix Methods Res 2017; 11: 291–307.

[11] Guetterman TC , Fetters MD , Creswell JW. Integrating quantitative and qualitative results in health science mixed methods research through joint displays. Ann Fam Med 2015; 13: 554–61. 26553895 10.1370/afm.1865 PMC4639381

[12] McCrudden MT , Marchand G , Schutz PA. Joint displays for mixed methods research in psychology. Meth Psychol 2021; 5: 100067.

[13] Fisher RA. Statistical methods for research workers. In Breakthroughs in Statistics (eds S Kotz, NL Johnson): 66–70. Springer, 1992.

[14] Welch BL. The generalisation of student’s problems when several different population variances are involved. Biometrika 1947; 34: 28–35. 20287819 10.1093/biomet/34.1-2.28

[15] R Core Team. R: A Language and Environment for Statistical Computing. R Foundation for Statistical Computing, 2021 (www.R-project.org/).

[16] Wickham H. ggplot2: Elegant Graphics for Data Analysis. Springer-Verlag, 2016.

[17] Ritchie J , Lewis J , Nicholls CM , Ormston R. Qualitative Research Practice: A Guide for Social Science Students and Researchers. SAGE Publications, 2013.

[18] Palinkas LA , Horwitz SM , Green CA , Wisdom JP , Duan N , Hoagwood K. Purposeful sampling for qualitative data collection and analysis in mixed method implementation research. Adm Policy Ment Health 2015; 42: 533–44. 24193818 10.1007/s10488-013-0528-y PMC4012002

[19] Malterud K , Siersma VD , Guassora AD. Sample size in qualitative interview studies: guided by information power. Qual Health Res 2016; 26: 1753–60. 26613970 10.1177/1049732315617444

[20] QSR International Pty Ltd. *NVivo (released in March 2020)*. Lumivero, 2020 (www.lumivero.com).

[21] Boeije H. A purposeful approach to the constant comparative method in the analysis of qualitative interviews. Qual Quan 2002; 36: 391–409.

[22] Ryland H , Davies L , Kenney-Herbert J , Kingham M , Deshpande M. Advancing research in adult secure mental health services in England. Med Sci Law 2022; 62: 225–9. 34907815 10.1177/00258024211066981 PMC9198389

[23] Kappen TH , Vergouwe Y , van Wolfswinkel L , Kalkman CJ , Moons KGM , van Klei WA . Impact of adding therapeutic recommendations to risk assessments from a prediction model for postoperative nausea and vomiting. Br J Anaesth 2015; 114: 252–60. 25274048 10.1093/bja/aeu321

[24] Miller IW , Camargo Jr CA , Arias SA , Sullivan AF , Allen MH , Goldstein AB , et al. Suicide prevention in an emergency department population: the ED-SAFE study. JAMA Psychiatry 2017; 74: 563–70. 28456130 10.1001/jamapsychiatry.2017.0678 PMC5539839

[25] Troquete NA , van den Brink RH , Beintema H , Mulder T , van Os TWDP , Schoevers RA , et al. Risk assessment and shared care planning in out-patient forensic psychiatry: cluster randomised controlled trial. Br J Psychiatry 2013; 202: 365–71. 23520222 10.1192/bjp.bp.112.113043

[26] Greenhalgh T , Papoutsi C. Studying complexity in health services research: desperately seeking an overdue paradigm shift. BMC Med 2018; 16: 95. 29921272 10.1186/s12916-018-1089-4 PMC6009054

[27] Petticrew M. When are complex interventions ‘complex’? When are simple interventions ‘simple’? Eur J Public Health 2011; 21: 397–8. 21771736 10.1093/eurpub/ckr084

[28] Skivington K , Matthews L , Simpson SA , Craig P , Baird J , Blazeby JM , et al. A new framework for developing and evaluating complex interventions: update of Medical Research Council guidance. BMJ 2021; 374: n2061. 34593508 10.1136/bmj.n2061 PMC8482308

[29] Craig P , Dieppe P , Macintyre S , Michie S , Nazareth I , Petticrew M. Developing and evaluating complex interventions: the new Medical Research Council guidance. BMJ 2008; 337: a1655. 18824488 10.1136/bmj.a1655 PMC2769032

[30] Cavanagh J , Deligianni F , Fenton S-JH , Gkoutos GV , Lee R , Leighton SP , et al. Prediction models in first-episode psychosis: systematic review and critical appraisal. Br J Psychiatry 2022; 220: 179–91. 35067242 10.1192/bjp.2021.219 PMC7612705

[31] Oliver D , Arribas M , Perry BI , Whiting D , Blackman G , Krakowski K , et al. Using electronic health records to facilitate precision psychiatry. Biol Psychiatry 2024; 96: 532–42. 38408535 10.1016/j.biopsych.2024.02.1006

[32] Damschroder LJ , Reardon CM , Widerquist MAO , Lowery J. The updated Consolidated Framework for Implementation Research based on user feedback. Implement Sci 2022; 17: 75. 36309746 10.1186/s13012-022-01245-0 PMC9617234

