# Supplementary material for: Use of a violence risk prediction tool (Oxford Mental Illness and Violence) in early intervention in psychosis services: mixed methods study of acceptability, feasibility and clinical role
Source: Br J Psychiatry. 2025 Mar 20;228(2):140–9. doi: 10.1192/bjp.2024.293 (PMC12823449; doi:10.1192/bjp.2024.293)

**Use of a violence risk prediction tool (Oxford Mental Illness and Violence) in early intervention in psychosis services: mixed methods study of acceptability, feasibility and clinical role**

**Supplementary Material**

**Supplement 1**

OxMIV assessments completed over 12 months. Blue lines indicate timing of repeat promotion of project in team meetings.

**
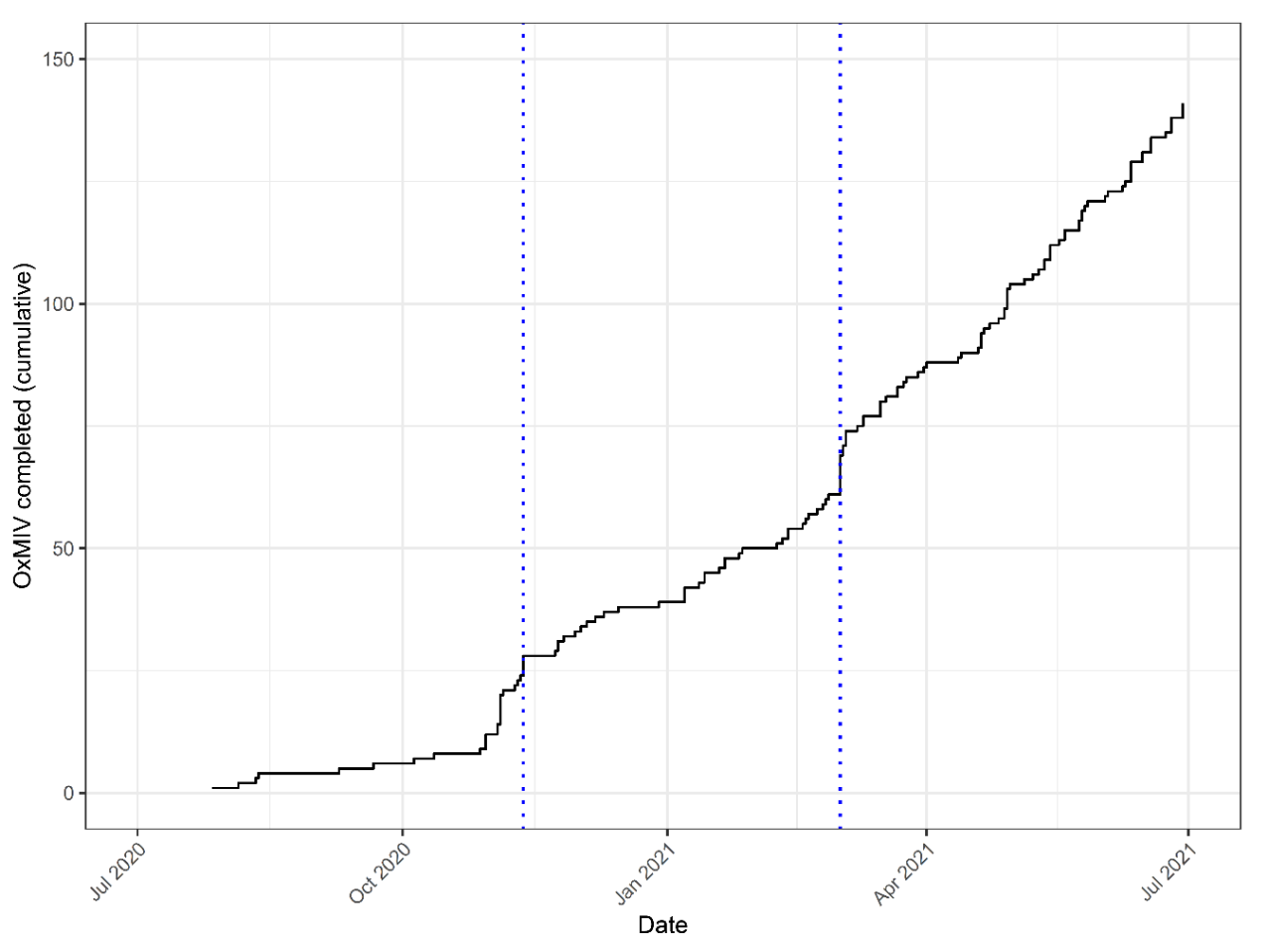
**

**Supplement 2**

Summary characteristics of sample of clinicians (multidisciplinary healthcare professionals) included in qualitative interviews.

| **Clinician characteristics** | | | **N** |
| --- | --- | --- | --- |
| **Sex** | Male | 8 | |
|  | Female | 12 | |
| **Age** | 21-30 | 3 | |
|  | 31-40 | 8 | |
|  | 41-50 | 3 | |
|  | >50 | 6 | |
| **Professional background** | Medic | 5 | |
|  | Occupational Therapist | 3 | |
|  | Social Worker | 4 | |
|  | Community Psychiatric Nurse | 6 | |
|  | Psychologist | 1 | |
|  | Other | 1 | |
| **Years working in mental health** | 0-5 | 6 | |
|  | 6-10 | 3 | |
|  | 11-20 | 5 | |
|  | >20 | 6 | |

**Supplement 3**

Themes developed across clinician interviews and exemplar quotes (in addition to those provided in main manuscript).

| **Themes** | **Exemplar quotes** |
| --- | --- |
| **1.** Clinical utility and impact on practice | *I've done quite a lot on risk factors for…offending…so I've got the knowledge there but it just kind of wasn’t there. So what it’s done* [OxMIV] *is it’s brought it to my fingertips a bit more. And made it…more accessible…more there in the room, I guess… I'm more aware of it, rather than it being…an abstract concept of what risk might be.* (P01, Psychiatrist) |
| **2.** Acceptability of the required clinical information | *I quite liked the way the setup intuitively made sense…and…the questions I was being asked…in my head…I could deduce why those questions would be …related to the overall risks. So that…was nice, I enjoyed using it.* (P14, Other MH Professional) |
| **3.** Practical usability and functionality within the Electronic Health Record | *…as soon as you have to click another box, and I know it doesn’t seem like much but… there is so much stuff… There is so much stuff and we’re asking people to do more things all the time… And then you’ll have a push because, you know somebody somewhere in data analysis have realised we’re not* [recording X] *status and so then you get an email round “Right a big push on* [X] *status.” You know so everyone’s got to click on again and… click, click, click.* (P13, Psychologist) |
| **4.** Interpretation of output | *I'm surprised about the real jump in some people. So, lots of people are very low risk. And then suddenly you just have some young guys who just have very high risk. And I guess that’s the case, isn’t it…that’s what’s it like in the general population. Most people do have very…low risk. If you’re a young male using drugs and alcohol and… you’re a bit paranoid, then actually you are [higher risk].* (P01, Psychiatrist) |
| **5.** Alignment and integration with clinical judgement |  |
| **6.** Positioning within the team and clinical pathway | *I think…it’s definitely quite good to have it as part of the assessment process, because ...we have quite a few tick boxes anyway, for different tabs. So…it’s quite easy to remember…to do it at that stage, so I think it’s quite good having it integrated into that process.* (P14, Psychiatrist) |
| **7.** Barriers to integration and adoption | *I suppose things that would prevent me* [using OxMIV in the future] *are it’s another form to fill in. We obviously have several forms already…and… it’s a bit of an add-on…or at least for me it didn’t feel like it would be pertinent for everyone…to have this tool…filled in. So, I can see how in a forensic setting…it would feel much more part of your bread and butter…rather than an add-on. So, I suppose…where it fits in the service and the patient population will vary.* (P11, Psychiatrist)  *…we haven’t had this previously and…implementing that change of mindset for me* [was a challenge] *and maybe just the title, the fact that it’s got violence in the title. I wonder whether that could make people think well that’s not applicable to me because my client isn’t violent.* (P03, Occupational Therapist) |
| **8.** Stigma and labelling |  |
| **9.** Discussion and collaboration with patients | *It’s quite a gentle tool isn’t it? …I think if I had somebody come out…higher risk I’d have been able to go through the tool with them and say look we’ve looked at this and this indicates the possibility and now what we’re going to do is look at how we can reduce this for you. How we can make it manageable… Doesn’t mean to say it’s going to happen, but we’ve got to…formulate something. If somebody’s risk is higher it would encourage you, it would drive your interventions wouldn’t it, it would be something that you would use to...reduce the risk.* (P02, Community Psychiatric Nurse)  *…if you’re doing a risk assessment then you're already starting to…classify levels of risk and whether you…do it in the OxMIV way…with some numbers or you don’t…I don’t, personally…think it makes that much difference and… what we’re trying to do is…to be…as aware as possible of levels of risk so that we can help manage them…that’s potentially very much in the patient’s benefit if we can help them…manage their risks and not end up in trouble…then that’s gonna help them as well as others.”* (P15, Psychiatrist)  *…what it* [OxMIV] *has helped…is to have the discussion…about what they consider might be the triggers. And that I think that has been helpful…in as much as they’ve been able to associate the acts of violence that they were involved with and that… substances…were a major contributory factor. So I think it’s opened up the opportunity to look at that in the round and say these are things that…are having a very negative effect on your behaviour if you continue to use these substances or in the way that you have. So I think it’s helpful to be able to have that conversation...* (P07, Social Worker) |
| **10.** Attitudes towards linked interventions | *…I mean ultimately the aim is about managing risk, isn’t it, I think? And…the first bit is assessing and then the second bit is, “Well, what can we do?” And…in some ways it feels like the second bit is harder, to…come up with a plan or figure out what would be good thing to do. So…that could be a very helpful thing to have some suggestions. But then…it would need to be viewed as suggestions rather than, you know, “You must do this or that.” But…at least there’d be things for consideration, wouldn’t there…? So yeah… I’d…view that as a…helpful direction…* (P15, Psychiatrist) |

**Supplement 4**

Summary characteristics of included patient and carer sample.

| **Participant ID** | **Role** | **Sex** | **Age bracket** |
| --- | --- | --- | --- |
| P1 | Patient | Female | 51-60 |
| P2 | Carer (parent) | Female | 51-60 |
| P3 | Carer (parent) | Female | 61-70 |
| P4 | Patient | Male | 21-30 |
| P5 | Patient | Male | 51-60 |
| P6 | Patient | Female | 31-40 |
| P7 | Patient | Female | 21-30 |
| P8 | Patient | Male | 21-30 |
| P9 | Carer (spouse) | Female | 51-60 |
| P10 | Patient | Female | 21-30 |
| P11 | Patient | Male | 21-30 |
| P12 | Patient | Male | 41-50 |

**Supplement 5**

1.
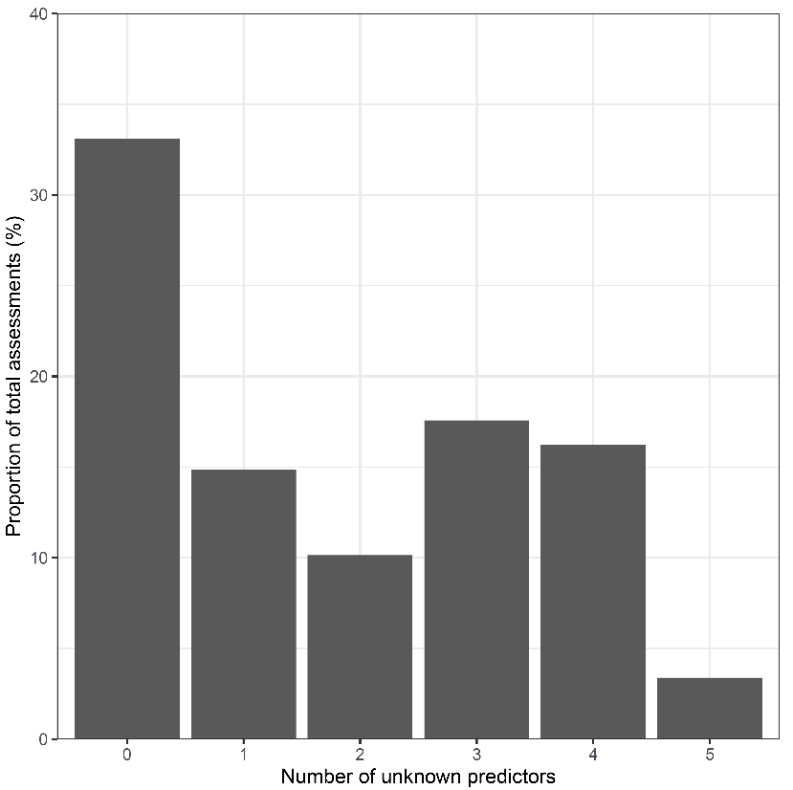
Proportion of 141 OxMIV assessments completed with 0, 1, 2, 3, 4 or 5 total missing predictors.
2. Proportion of "unknown" scores for each predictor in 141 OxMIV assessments.


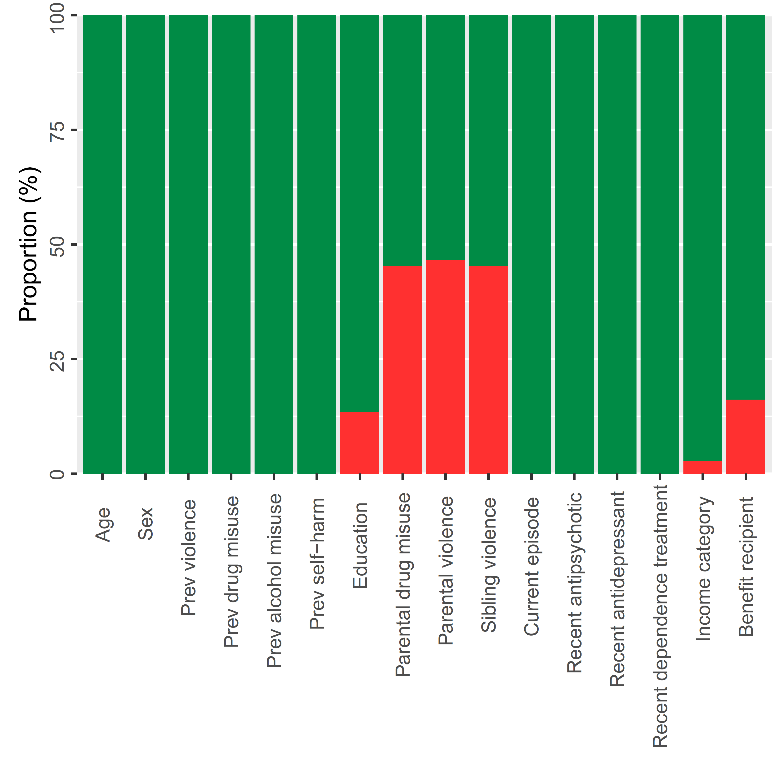

Supplement: Whiting et al. supplementary material [file S0007125024002939sup001.docx]
